# Editorial to “comparison of ex vivo lesion formation for two adjacent radiofrequency applications with very‐high‐power short‐duration in various inter‐lesion times”

**DOI:** 10.1002/joa3.13202

**Published:** 2024-12-10

**Authors:** Koji Fukuzawa, Mitsuru Takami, Kimitake Imamura

**Affiliations:** ^1^ Section of Arrhythmia, Division of Cardiovascular Medicine, Department of Internal Medicine Kobe University Graduate School of Medicine Kobe Japan; ^2^ Division of Cardiovascular Medicine, Department of Internal Medicine Kobe University Graduate School of Medicine Kobe Japan

**Keywords:** atrial fibrillation, inter‐lesion time, radiofrequency ablation, resistive and conductive heating, very‐high‐power short‐duration ablation

Durable pulmonary vein isolation is the cornerstone of radiofrequency (RF) catheter ablation of atrial fibrillation (AF). Sufficient RF energy deliveries at each target point are essential, and “sequential” energy applications with a close inter‐lesion distance would be one of the solutions for a first‐pass isolation without any gaps. What are the differences in the lesion formation created by sequential or point‐by‐point RF application maneuvers? Regarding this issue, Dr. Hanaki and his colleagues reported important observations about both the efficacy and safety when delivering very high‐power short‐duration (vHPSD, 90‐watt power setting for 4 s) RF applications by focusing on the inter‐lesion “time”.^1^ Various methods to achieve uniform, transmural lesions during pulmonary vein isolation while minimizing collateral damage have been studied, such as the RF power settings, contact force with the tissue, and application duration, and those parameters are integrated into the index to detect the lesion formation. A close inter‐lesion distance of less than 6 mm with adequate index values promises a gap‐less isolation line. In addition to those previous pieces of knowledge, the authors gave us an important awareness of the inter‐lesion time.

According to the authors' report, the lesion depth between sequential vHPSD RF applications (intermediate lesion depth) with a shorter inter‐lesion time of less than 20 s was 3 mm, which was comparable to the lesion depth with a single vHPSD RF application and deeper than that with sequential applications with an inter‐lesion time of 60 s. RF ablation lesions result from thermal injury that occurs in 2 consecutive phases: resistive and conductive heating phases.^2^ The vHPSD ablation system was developed to create a uniform, transmural lesion while avoiding collateral damage. Conceptually, it was thought that vHPSD ablation is mainly based on resistive heating with a minimum impact of conductive heating (thermal latency).^2^ Conversely, Dr. Nakagawa stated that the majority of effective tissue heating and, thereby, lesion formation with vHPSD RF applications occur due to conductive tissue heating after termination of the RF delivery.^3^ The latter theory can explain the authors' results of the deeper intermediate lesion depth by sequential applications with a shorter time interval. Sequential RF applications during the persistence of thermal latency in the surrounding tissue can cause an additional impact on lesion formation of the intermediate lesion.

A uniform and adequate transmural lesion with vHPSD ablation promises a first‐pass isolation with a short procedure time. However, the left atrial wall has a thickness of 1–3 mm and is not uniform depending on the region and patient. A uniform RF application throughout the isolation line targeting a lesion depth of 3 mm can cause both insufficient RF applications with thicker walls and excessive applications with thinner walls. As described by the authors, a one‐size‐fits‐all RF application maneuver throughout the isolation line may cause unintended deeper thermal effects on the neighboring organs and tissues, such as the esophagus and phrenic nerve. We should be aware that unintentional deeper lesions can be created by sequential applications with a short inter‐lesion time. Another investigator also reported that double vHPSD RF applications without resting can create a deeper lesion.^4^ Based on the authors' and previous findings, we might be able to control the lesion depth at the target and intermediate lesions while adjusting the inter‐lesion time. Theoretically, short‐coupled sequential applications would be desirable for thicker muscles, and point‐by‐point applications with an adequate inter‐lesion time or stepping‐stone‐like application for thinner muscles regarding both the effectiveness and safety of the pulmonary vein isolation. However, most importantly, when the authors' findings are applied in clinical practice, some limitations of the experiment using an ex vivo dead swine heart model must be considered, as the authors described. Particularly for safety, sequential vHPSD RF applications without a resting time would cause excessive tissue heating, potentially increasing steam pops.

Now, pulsed field ablation (PFA) systems have been launched in Japan. In Europe and North America, PFA became available several years before, and PFA has become a major energy source for the 1st session of AF ablation. How can we apply the authors' findings in the PFA era? However, PFA is not a panacea for all arrhythmia treatments. PFA on the mitral isthmus and right isthmus can cause coronary spasms, leading to life‐threatening complications. Further, which energy sources should we use in the second and third sessions of AF or atrial tachycardia ablation? For catheter‐based ablation therapy, there will remain considerable opportunities to take advantage of RF energy with detailed mapping. A similar sequential RF application effect would be present for normal (<30 watts) or middle‐power ablation (30–50 watts). With a greater impact of conductive heating with normal or middle‐range power, the additional impact of sequential applications would have appeared. Moreover, the authors' findings and valuable pieces of knowledge can be applied for ventricular arrhythmia RF ablation targeting a deeper lesion creation with intentional sequential applications and careful attention to steam pops.

Finally, the reviewer appreciates the authors' valuable contribution to understanding the biophysics of RF applications with vHPSD and the different inter‐lesion times. To apply the authors' findings in the clinical setting, it is essential to have an excellent technique for manipulating the catheter tip to the next target site within a short time. We, electrophysiologists, should maintain and improve our skills and knowledge of RF catheter ablation and pass that on to future generations even in the upcoming pulse field one‐shot ablation‐dominant era. “Pitch Clock” for the efficacies and safety of RF ablation.

## CONFLICT OF INTEREST STATEMENT

The authors have nothing to report.

## FUNDING INFORMATION

The Section of Arrhythmia (Kobe University Graduate School of Medicine) is financially supported by an endowment from Abbott Japan, Boston Scientific Japan, and Medtronic Japan. KF and KI belong to the Section, and KF receives a scholarship donation from Biotronik Japan. The authors report no relationships relevant to the contents of this manuscript.
